# *Arabidopsis* RIBA Proteins: Two out of Three Isoforms Have Lost Their Bifunctional Activity in Riboflavin Biosynthesis

**DOI:** 10.3390/ijms131114086

**Published:** 2012-10-31

**Authors:** Hanna-Maija Hiltunen, Boris Illarionov, Boris Hedtke, Markus Fischer, Bernhard Grimm

**Affiliations:** 1 Institute of Biology/Plant Physiology, Humboldt University Berlin, Philippstr. 13, Building 12, D-10115 Berlin, Germany; E-Mails: hannamaijahiltunen@gmail.com (H.-M.H.); boris.hedtke@rz.hu-berlin.de (B.H.); 2 Hamburg School of Food Science, Institute of Food Chemistry, University Hamburg, Grindelallee 117, 20146 Hamburg, Germany; E-Mails: illarion@chemie.uni-hamburg.de (B.I.); markus.fischer@chemie.uni-hamburg.de (M.F.)

**Keywords:** riboflavin, flavo-coenzyme, bifunctional enzyme, Arabidopsis, FAD and FMN

## Abstract

Riboflavin serves as a precursor for flavocoenzymes (FMN and FAD) and is essential for all living organisms. The two committed enzymatic steps of riboflavin biosynthesis are performed in plants by bifunctional RIBA enzymes comprised of GTP cyclohydrolase II (GCHII) and 3,4-dihydroxy-2-butanone-4-phosphate synthase (DHBPS). Angiosperms share a small *RIBA* gene family consisting of three members. A reduction of AtRIBA1 expression in the *Arabidopsis rfd1*mutant and in RIBA1 antisense lines is not complemented by the simultaneously expressed isoforms AtRIBA2 and AtRIBA3. The intensity of the bleaching leaf phenotype of RIBA1 deficient plants correlates with the inactivation of AtRIBA1 expression, while no significant effects on the mRNA abundance of AtRIBA2 and AtRIBA3 were observed. We examined reasons why both isoforms fail to sufficiently compensate for a lack of RIBA1 expression. All three RIBA isoforms are shown to be translocated into chloroplasts as GFP fusion proteins. Interestingly, both AtRIBA2 and AtRIBA3 have amino acid exchanges in conserved peptides domains that have been found to be essential for the two enzymatic functions. *In vitro* activity assays of GCHII and DHBPS with all of the three purified recombinant AtRIBA proteins and complementation of *E. coli ribA* and *ribB* mutants lacking DHBPS and GCHII expression, respectively, confirmed the loss of bifunctionality for AtRIBA2 and AtRIBA3. Phylogenetic analyses imply that the monofunctional, bipartite RIBA3 proteins, which have lost DHBPS activity, evolved early in tracheophyte evolution.

## 1. Introduction

Riboflavin (vitamin B2) is synthesized *de novo* in plants, fungi, archaea and numerous bacteria, while animals depend on dietary supply [1]. Riboflavin is the precursor for the synthesis of flavin mononucleotide (FMN) and flavin adenine dinucleotide (FAD), which are essential cofactors for numerous enzymes (e.g., dehydrogenases, oxidases, reductases) that participate in one- and two-electron oxidation-reduction processes critical for major metabolic pathways in all organisms. In plants, these cofactors are required for photosynthesis, mitochondrial electron transport, fatty acid oxidation, photoreception, DNA repair, metabolism of other cofactors and biosyntheses of numerous secondary metabolites [2,3].

**Figure 1:**
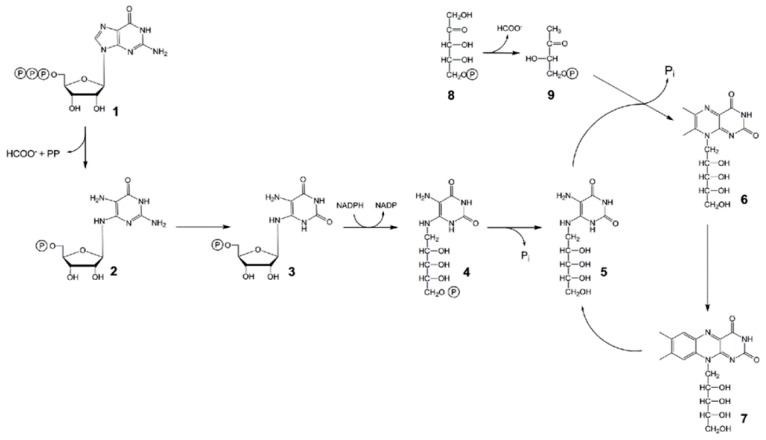
Biosynthesis of riboflavin. Riboflavin biosynthesis is initiated by the enzymes GTP cyclohydrolase II (GCHII) and 3,4-dihydroxy-2-butanone-4-phosphate synthase (DHBPS) converting GTP (**1**) into 2,5-diamino-6-ribosylamino-4(3*H*)-pyrimidinone 5′-phosphate (**2**) and ribulose-5-phosphate into 3,4-dihydroxy-2-butanone 4-phosphate (**9**), respectively. Both **2** and **9** are the first committed substrates of the riboflavin biosynthetic pathway. Following the biosynthetic pathway to riboflavin (**7**), **2** is consecutively modified to 5-amino-6-ribosylamino-2,4(1*H*,3*H*)-pyrimidine 5′-phosphate (**3**), 5-amino-6- ribitylamino-2,4(1*H*,3*H*)-pyrimidinedione 5′-phosphate (**4**) and 5-amino-6-ribitylamino- 2,4(1*H*,3*H*)-pyrimidinedione (**5**) by deaminase, reductase and phosphatase reactions, respectively. At present, it is still not clear if one specific phosphatase or several less specific enzymes are implemented in the dephosphorylation of **4. 5** is condensed with **9** by the enzyme lumazine synthase giving rise to 6,7-dimethyl-8-ribityllumazine (**6**). Finally, riboflavin synthase catalyzes a dismutation reaction of two molecules of **6** to form **7** yielding **5** as a byproduct, which again serves as substrate for lumazine synthase.

The riboflavin biosynthesis pathway is similar in plants, yeast and bacteria [1,4]. Riboflavin is synthesized by a series of seven distinct enzymatic reactions from GTP (**1** in Figure 1) and ribulose 5-phosphate (**8**), and then phosphorylated to FMN and adenylated to FAD. The reactions of riboflavin biosynthesis are described in Figure 1.

Detailed information is available about biochemistry and the regulation of vitamin B_2_ and flavin nucleotide biosynthesis in bacteria and fungi. Although plants are a major source of riboflavin for animals, only a few studies were dedicated to riboflavin biosynthesis in plants, its regulation and subcellular localization. Thus, early studies reported an activity converting **6** to **7** in leaves [5] and a partially purified enzyme from spinach [6]. Then, based on sequence similarity to their microbial homologs, several cDNA sequences of the pathway have been cloned from plants [3,7–11] providing strong evidence that riboflavin biosynthesis proceeds through almost the same steps in plants, fungi and bacteria.

Based on experimental and bioinformatic evidence, the enzymes of plant riboflavin biosynthesis are considered to reside in plastids [8–11]. In continuation of the pathway, phosphorylation of riboflavin to FMN and subsequent adenylation to FAD are catalyzed by the enzymes riboflavin kinase and FAD synthetase, respectively, in the presence of ATP and Mg^2+^[12–14]. These proteins have been found in plants to be not solely located in plastids [14]. Nevertheless, the subcellular distribution of enzymes catalyzing flavin nucleotide biosynthesis and hydrolysis is neither completely understood in plants nor other eukaryotic organisms.

The two initial reactions in riboflavin biosynthesis, starting at substrates **1** and **9** (Figure 1), respectively, are accomplished in several eubacteria, including *E. coli*, by genes designated *ribA* and *ribB* which encode monofunctional GCHII and DHBPS proteins, respectively [15,16]. However, other prokaryotes, such as *Bacillus subtilis* or cyanobacteria, produce a bifunctional RibA protein consisting of a DHBPS region in its *N*-terminal and a GCHII region in its *C*-terminal part [15].

In addition to the known *Arabidopsis RIBA* gene (hereafter designated *AtRIBA1*) [10], two additional genes encoding putatively bifunctional RIBA proteins (*AtRIBA2* and *AtRIBA3*) are present in the *A. thaliana* genome. In two previous reports, the AtRIBA1 mutant *rfd1* has been characterized by a dramatic down-regulation of *AtRIBA1* expression and reduced flavin contents *in planta*[17,18]. Hence, the homologous genes *AtRIBA2* and *AtRIBA3* were not able to complement the loss of *AtRIBA1*. A detailed look at the amino acid sequences of AtRIBA2 and AtRIBA3 revealed that several conserved amino acid residues are missing, either in the RIBA2 or the RIBA3 sequence, which are considered to be essential for the catalytic properties of enzymes [17]. To gain further insights into the functions of the AtRIBA proteins we performed expression studies of the three *RIBA* genes, assayed the enzymatic activities of the three recombinant AtRIBA isoforms *in vitro* and complemented *E. coli* ribA and ribB knock-out mutants.

## 2. Results

### 2.1. Expression Patterns of AtRIBA Genes

**Figure 2:**
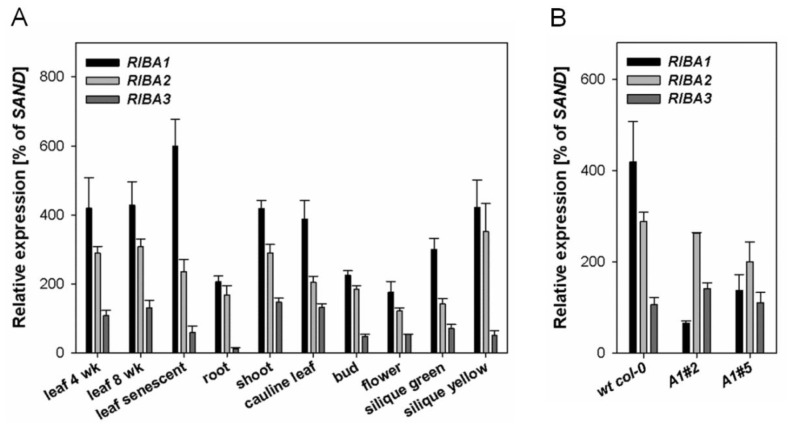
qPCR analyses of *AtRIBA* genes. (**A**) transcript accumulation of *Arabidopsis RIBA* genes was examined in different tissue types and (**B**), in two representative AtRibA1 antisense lines with intermediate (*A1#5*) and strong bleaching phenotype (*A1#2*), respectively. Expression was calculated relative to mRNA levels of SAND (At2g28390).

To examine the metabolic impact of the three RIBA isoforms identified in *A. thaliana*, the relative transcript amounts of the three *AtRIBA* homologs were assessed using qRT-PCR analyses in different tissues and developmental stages of wild-type plants. Transcripts of the three *RIBA* homologs accumulate in all analyzed tissues, but transcript levels were different (Figure 2A). The accumulation of *AtRIBA1* mRNA exceeded the transcript levels of *AtRIBA2* and *AtRIBA3* in all organs and developmental stages analyzed. However, the widely parallel accumulation of *AtRIBA* transcripts does not indicate a strong tissue specificity of gene expression of single members of the *AtRIBA* gene family.

### 2.2. Downregulation of *AtRIBA1* Causes a Bleached Phenotype

**Figure 3:**
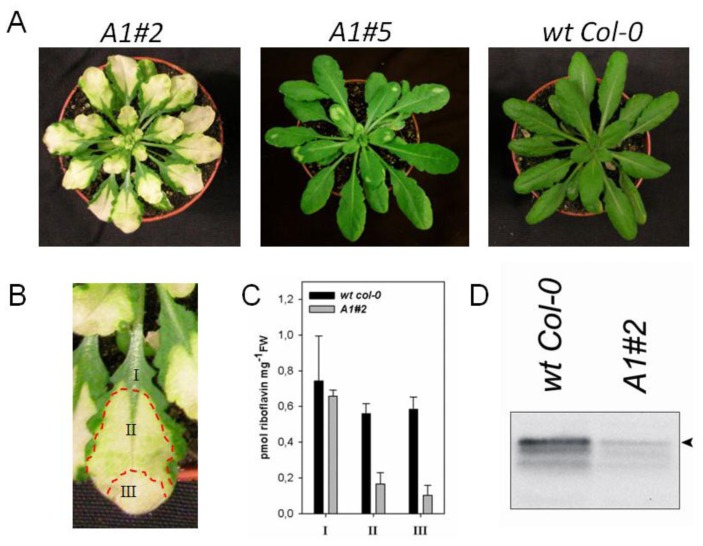
Phenotype of antisense *AtRIBA1* plants. (**A**) Different degrees of bleaching are the result of *AtRIBA1* antisense expression. Plants displaying a moderate antisense phenotype (left panel) start to bleach partially at the tip of leaves in the rosette stage, while individuals with a stronger reduction in *AtRIBA1* transcript amounts display white inner rosette leaves and shoot apical meristem (middle). A *Arabidopsis thaliana* ecotype *Columbia* (*Col-0*) wild-type plant of the same age is depicted in the right panel. (**B**) For HPLC analyses, leaves of line *A1#2* were harvested and dissected as indicated. I: green, II: medium, III: white pigmentation. Reference samples were collected from comparable regions of wild-type plants. (**C**) Leaf regions depicted in (**B**) were subjected to flavin extraction and analyzed for the content of riboflavin using HPLC. (**D**) Immunodetection of AtRIBA protein in whole leaf extracts of line *A1#2* and wild-type *Col-0* control using anti-RIBA1 specific antiserum. Both samples represent identical fresh weight amounts. Although the antiserum recognizes all three AtRIBA isoforms, the upper band (arrow head) was shown to represent AtRIBA1 by an analysis of overexpressing lines (data not shown).

A strong down-regulation of the *AtRIBA1* gene expression in the *Arabidopsis rfd1* mutant correlated with a bleached phenotype [18]. Seedlings were unable to grow photoautotrophically on soil and their growth was abandoned in sugar-supplemented media after several weeks. Interestingly, the bleaching phenotype of the seedlings is not counterbalanced by the simultaneous *AtRIBA2* and *AtRIBA3* expression, which remains at wild-type levels in *rfd1*[17]. For a gradual reduction of riboflavin biosynthesis, several transgenic lines with *AtRIBA1* antisense RNA expression under control of the CaMV 35S promoter were generated (Figure 3A). *RIBA* mRNA levels were determined in rosette leaves of two representative AtRIBA antisense lines (*A1#2* and *A1#5*) displaying different degrees of pigment deficiency. While the intensity of the phenotype in the two selected lines correlates with reduced *AtRIBA1* transcript levels, *AtRIBA2* and *AtRIBA3* are expressed at levels comparable to wild-type tissue (Figure 2B). Hence, the expression of the latter isoforms does not prevent the deficiency in riboflavin biosynthesis and the decline of *AtRIBA1* expression in *Arabidopsis* was not compensated for at the transcriptional level by modified activity of the homologous genes.

The phenotypic alterations of individuals with a severe phenotype, including line *A1#2*, were compared between three leaf sections. While the leaf base (Sample I in Figure 3B,C) was similar to a wild type, a progressive loss of pigmentation was observed towards the leaf tip (samples II and III). In these leaf sections, a gradual reduction of riboflavin (Figure 3C) was observed. AtRIBA1 protein levels were strongly decreased in *A1#2* leaves (Figure 3D), agreeing with the observed reduction in mRNA amounts.

### 2.3. Subcellular Localization of AtRIBA Proteins

Since all three AtRIBA genes are constantly expressed, the observed AtRIBA1 depletion phenotype of *rfd1*[18] and the antisense lines depicted in Figure 3A could be explained by spatial separation of AtRIBA proteins in different subcellular compartments.

**Figure 4:**
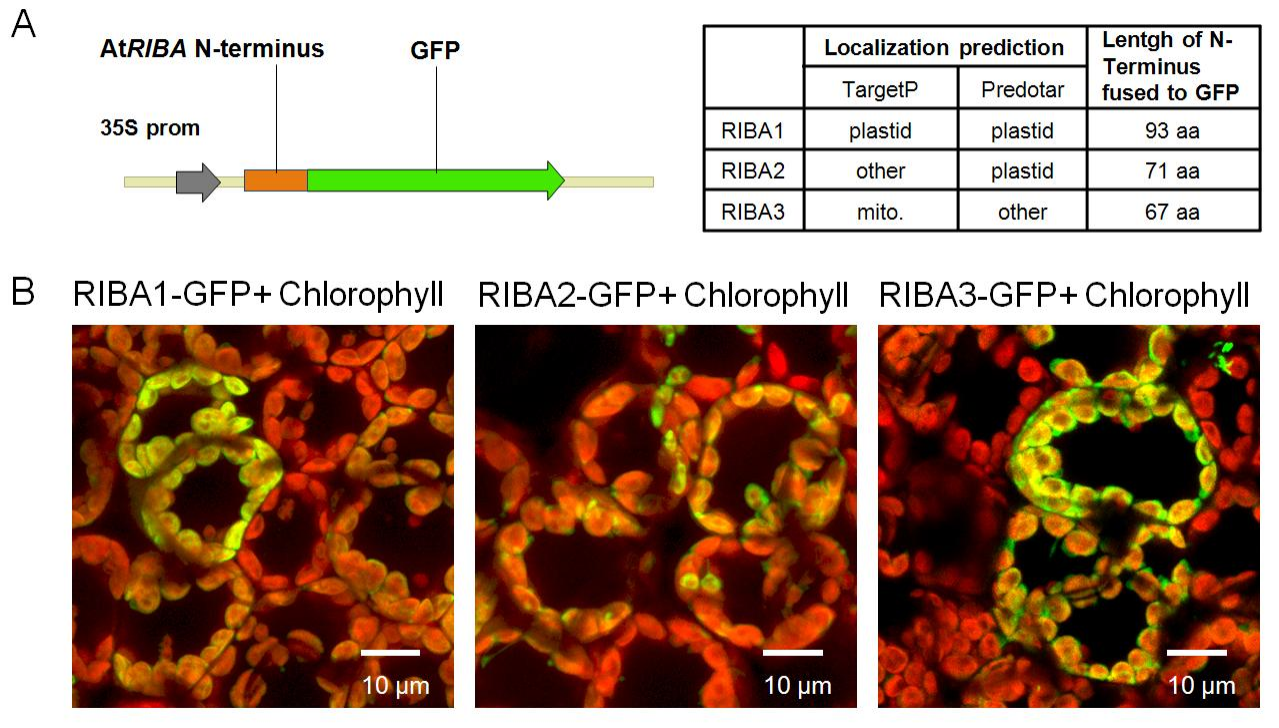
Green fluorescent protein (GFP) localization experiments. (**A**) The amino terminal sequences comprising the putative transit peptides were translationally fused to the *N*-terminus of GFP. Targeting properties were predicted using TargetP and Predotar. The lengths of the AtRIBA aminotermini tested experimentally are indicated. (**B**) RIBA-GFP fusions were expressed transiently in *Agrobacterium*-infiltrated *Nicotiana benthamiana* leaves and visualized in mesophyll cells using Confocal Laser Scanning Microscopy. The three panels show merged images of GFP and chlorophyll fluorescence indicating that green fluorescence localizes within the plastid compartment for all three RIBA-GFP fusion constructs investigated.

To address the localization of the three AtRIBA proteins, iPSORT [19], TargetP [20] and Predotar [21] algorithm were used for targeting predictions of their subcellular localization (Figure 4A). The prediction hinted at the existence of an *N*-terminal RIBA transit peptide and either a plastidic or mitochondrial localizations. We examined *in situ* targeting of the three RIBA homologs by CLSM-mediated visualization of transiently expressed green fluorescent protein (GFP) fusion proteins after transformation of *Nicotiana benthamiana* leaves. Based on predictions by TargetP, three different gene constructs encoding the putative transit peptides of AtRIBA1-3 were fused to 5′-end of the GFP-encoding sequence (Figure 4A). The co-localization of chlorophyll and GFP fluorescence observed for all three RIBA-GFP fusions (Figure 4B) clearly demonstrates that all AtRIBA *N*-termini contain plastid targeting signals.

### 2.4. *In vitro* Enzyme Assays with Recombinant AtRIBA Proteins

For detailed comparative characterization of AtRIBA enzymes *in vitro*, all three RIBA-encoding sequences were expressed in *E. coli*. The design of the artificial RIBA sequences included adaptation to *E. coli* codon usage and addition of an *N*-terminal His–tag (Data S1). All RIBA proteins specified by newly generated plasmid constructs lacked the plant-specific *N*-terminal sequence including the transit peptide. Thus, in comparison to the wild-type protein precursor sequences the recombinant proteins lacked the first 127 (AtRIBA1), 105 (AtRIBA2) or 100 amino acids (AtRIBA3), respectively (cf. alignment in Figure S1B in [17]).

**Figure 5:**
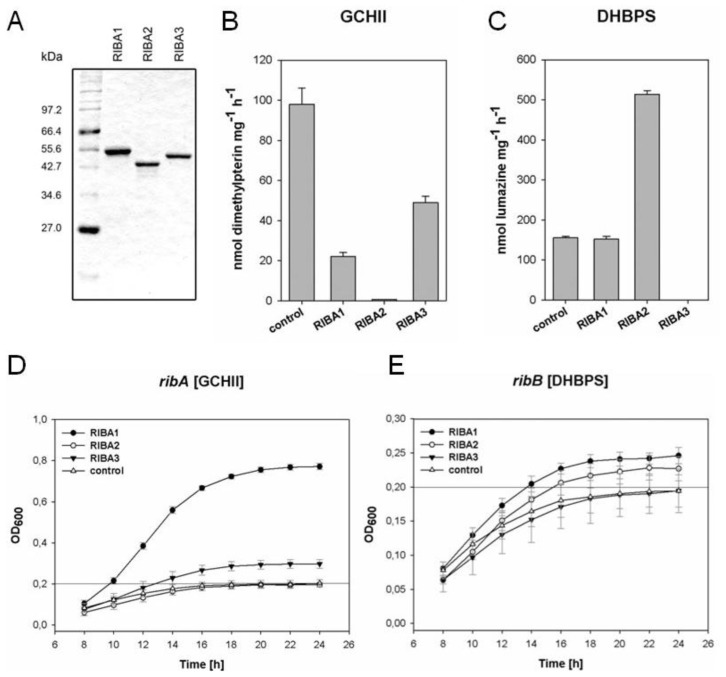
Enzymatic activities of AtRIBA proteins. (**A**–**C**) His-tagged *N*-terminally truncated AtRIBA proteins were overexpressed in *E. coli* and purified by FPLC. (**A**) Coomassie staining following SDS PAGE detects highly enriched recombinant *Arabidopsis* proteins in selected FPLC fractions. 0.75 μg of each recombinant RIBA protein were applied. The obtained fractions were assayed *in vitro* for enzymatic activity for GCHII (**B**) and DHBPS (**C**). In both assays a standard RIBA protein (RibA from *Bacillus subtilis*) was included as positive control. (**D**,**E**) *E. coli ribA* (**D**) and *ribB* (**E**) mutants were transformed with plasmids encoding the three *Arabidopsis* RIBA isoforms. Growth (OD_600_) of at least three independent cultures in liquid M9 minimal medium was monitored for 24 h; the initial absorption of the culture was subtracted. Empty vector pACYC184 was used as negative control; the maximum density reached by the control did not exceed an OD_600_ of 0.2 (grey line). Standard errors are indicated.

All three over-produced recombinant RIBA proteins were purified by FPLC using metal affinity chromatography. SDS polyacrylamide gel electrophoresis of the purified recombinant AtRIBA1-3 proteins confirmed the predicted molecular weights of 47.6, 42.3 and 46.5 kDa, respectively (Figure 5A). The purified AtRIBA proteins were assayed for GCHII and DHBPS activity *in vitro* (Figure 5B,C). A GCHII enzymatic function was clearly demonstrated for RIBA1 as well as for RIBA3, whereas RIBA2 did not display a detectable activity (Figure 5B). A DHBPS activity was determined for RIBA1 and RIBA2. Here, RIBA3 did not display a measurable DHBPS enzymatic function (Figure 5C). Taken together, the data obtained from *in vitro* assays clearly indicate that both recombinant AtRIBA2 and AtRIBA3 isoforms are able to carry out only one of two enzymatic activities of bifunctional RIBA proteins.

### 2.5. Complementation of Bacterial Mutants

In *E. coli*, *ribA* and *ribB* genes encode monofunctional GCHII and DHBPS enzymes, respectively. Corresponding *E. coli* mutant strains were employed to study complementation of respective enzymatic functions by RIBA1-3 *in situ* and corroborate the data obtained *in vitro.*

*AtRIBA* sequences designed for expression in *E. coli* (Data S1) were cloned into pACYC184 and transformed into *E. coli ribA* and *ribB* mutants, respectively. The vector pACYC184 was also used as blank control. We assayed the growth rate (OD_600_) of resulting *E. coli* transformants in M9 minimal medium liquid cultures (Figure 5D,E). Since *ribA* and *ribB* are riboflavin auxotrophs, precultures were grown in LB medium containing 0.4 g/L riboflavin. At OD_600_ of 0.6, the cells were pelleted, washed and resuspended in M9 minimal medium (OD_600_ = 0.1) and grown at 37 °C for 24 h.

Cultures of both *E. coli* mutants containing plasmid pACYC184 reached in M9 minimal medium a stationary phase at OD_600_ of approx. 0.2 (Figure 5D,E). Cultures of the *ribA* mutant (deficiency in GCHII) revealed an enhanced growth rate when transformed with RIBA1 or RIBA3 constructs, while the growth rate of *E. coli ribA* cells transformed with pACYC-AtRIBA2 did not differ from the blank control strain (Figure 5D). The culture of the DHBPS-deficient mutant strain *ribB* displayed an enhanced growth when transformed with either the RIBA1 or RIBA2 encoding plasmid. In contrast, pACYC-AtRIBA3 was not able to improve the growth rate in comparison to the empty control plasmid (Figure 5E).

## 3. Discussion

### 3.1. Consequences of AtRIBA1 Deficiency in Arabidopsis

Based on the description of *rfd1*[17,18], the specific function of the three homologous *AtRIBA* genes in *Arabidopsis* riboflavin biosynthesis was further investigated. Transgenic lines were generated that constitutively express *AtRIBA1* antisense RNA. These new lines displayed various degrees of a bleaching leaf phenotype (Figure 3A) occurring at different stages of plant development. An early bleaching of individual lines, like *A1#2*, phenotypically resembled *rfd1*. These lines were unable to survive under photoautotrophic conditions. The leaves of other transgenic lines including *A1#5* bleached later during plant development (Figure 3A). Here, the first leaves developed with wild-type like pigmentation, while new leaves in the *Arabidopsis* rosette stage turned white. The loss of pigmentation corresponds to inactivation of *AtRIBA1* expression as demonstrated by quantitative PCR analysis (Figures 2B and 3D).

Numerous *Arabidopsis* tissues were analyzed by qPCR to compare the abundance of *AtRIBA* mRNAs. The transcript profile of all three homologous *RIBA* genes (Figure 2A) reveals a largely constitutive expression with *AtRIBA1* being the most abundant transcript in all tested tissues. This implies that *AtRIBA2* as well as *AtRIBA3* are ubiquitously expressed and their expression has at least the potential to partially complement *AtRIBA1* deficiency. The transcript analysis of antisense lines (Figure 2B), however, demonstrates that there is no concerted transcriptional regulation of the *RIBA* gene family members in *Arabidopsis,* since the isogenes did not show altered transcript accumulation in AtRIBA1 antisense lines.

It could be speculated that the characteristic bleaching phenotype is explained by an insufficient dosage of expressed RIBA protein as the overall amount of *RIBA* transcripts and proteins in antisense plants is severely reduced (Figures 2B and 3C,D). Alternatively, the lack of complementation of AtRIBA1 deficiency might be caused by differences in subcellular localization or enzymatic functions among the homologs.

The localization of the RIBA isoforms was tested employing GFP fusions in transiently transformed *Nicotiana* leaf cells. All three AtRIBA proteins possess N-terminal sequences that exclusively direct GFP fusions to plastids (Figure 4). These findings agree with mass spectrometry data available for all three proteins from plastid proteome projects as summarized in the SubCellular Proteomic Database (SUBA, http://suba.plantenergy.uwa.edu.au/) [22] as well with data available for further enzymes of riboflavin synthesis (SUBA entries for At4g20960, At2g44050, At2g20690) and, thus, corroborates the assumption that plant riboflavin biosynthesis is exclusively localized in plastids [9]. In conclusion, the plastid localization of all three RIBA isoforms does not explain the inability of RIBA2 and RIBA3 to complement RIBA1 deficiency in *Arabidopsis*.

Differences in enzymatic activities of the bifunctional *A. thaliana* RIBA homologs have previously been suggested when the protein primary structures were compared with sequences of bacterial RibA proteins (Figure S1 in [17]). Amino acids known to be indispensable for either binding of zinc ions (*i.e.* for GCHII activity) or substrate binding and catalysis (*i.e.*, for DHBPS activity) are lacking in AtRIBA2 and AtRIBA3, respectively [23,24]. The recent characterization of a *N. benthamiana* RIBA homolog underlined the functional importance of specific residues in plant RIBA sequences [25].

### 3.2. Analyses of Enzymatic Activities of AtRIBA Isoforms

The present work aimed at comparatively examining the enzymatic activities of all three AtRIBA homologs by two alternative approaches. First, purified recombinant RIBA proteins were employed in *in vitro* assays (Figure 5A–C). Second, the complementation of *E. coli ribA* and *ribB* mutants substantiated the results *in situ* (Figure 5D,E). A *ribB* (DHBPS)-deficient strain showed improved growth characteristics when complemented with the AtRIBA1 and AtRIBA2 sequences. Although the complementation was only partial, the elevated growth rate was characteristic in comparison to that of the same mutant containing the AtRIBA3-expressing plasmid. The complementation of *ribA* deficient *E. coli* cells was more effective by AtRIBA1 than by AtRIBA3. However, the latter exceeded the effect of AtRIBA2 and the pACYC184 vector control.

In conclusion, both the *in vitro* and the *in situ* approaches confirmed the hypothesis that AtRIBA2 as well as AtRIBA3 represent only mono- instead of bifunctional enzymes for riboflavin biosynthesis: while AtRIBA2 is lacking GCHII activity, AtRIBA3 does not display DHBPS function.

### 3.3. RIBA Genes Lacking DHBPS Activity Evolved Early in Vascular Plant Phylogeny

The architecture of bifunctional enzymes has been suggested to favor the coordination of expression and catalysis [26]. GCHII and DHBPS are the first committed enzymes of a converged pathway, in which two molecules of the DHBPS product and one of GCHII (**9** and **2** in Figure 1) are required to finally synthesize riboflavin. Interestingly, *in vitro* enzyme assays revealed a two times higher activity of recombinant DHBPS compared to GCHII [10]. Hence, the expression of a fused DHBPS-GCHII protein could ensure catalysis of adequate amounts of reaction products required in the riboflavin biosynthetic pathway [26].

We make the point that the *Arabidopsis* genome contains two genes encoding bipartite proteins with only one enzymatic function in the riboflavin biosynthetic pathway. We assert, with our results, that expression of both monofunctional enzymes is insufficient for a replacement of bifunctional AtRIBA1. Different reasons may account for the lack of complementation by means of AtRIBA2 and AtRIBA3: their lower expression strength in comparison to AtRIBA1, steric hindrance of the two monofunctional proteins or an involvement of the latter in other metabolic pathways.

In addition, we can not entirely exclude potential mechanistic and structural functions of RIBA2 and RIBA3 in the formation of multienzymatic complexes in riboflavin biosynthesis. Thus, sequestration and protection of labile metabolic intermediates can improve metabolic channeling as demonstrated recently for bifunctional BIO3-BIO1 in *Arabidopsis* biotin synthesis [27].

**Figure 6:**
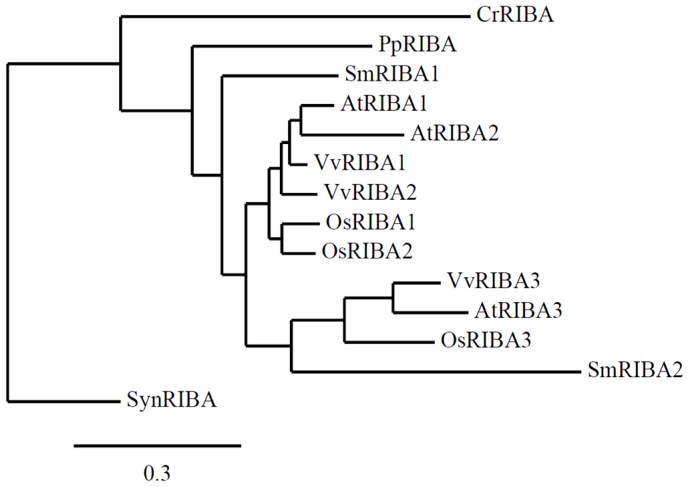
Phylogenetic tree of selected plant RIBA protein sequences. *RIBA* gene families identified in the angiosperm species *Arabidopsis thaliana* (At), *Vitis vinifera* (Vv) and *Oryza sativa* (Os), two RIBA proteins from a lycopodiophyte species (*Selaginella*, Sm), as well as single RIBA sequences from a moss (*Physcomitrella*, Pp), a green algae (*Chlamydomonas*, Cr), and a cyanobacterium (*Synechococcus*, Syn) are included in the analysis. Alignment (using MUSCLE 3.7 and Gblocks 0.91b), phylogenetic analysis (PhyML3.0 aLRT) and tree rendering (TreeDyn 198.3) were performed using the phylogeny resource (http://www.phylogeny.fr) [29], the tree was re-rooted using SynRIBA as outgroup. Full length RIBA amino acid sequences of the following species were used: *Arabidopsis thaliana* (accession nrs. NP_201235, NP_179831, NP_568913), *Chlamydomonas reinhardtii* (XP_001689850), *Oryza sativa* (NP_001047195, BAD09287, NP_001055757), *Physcomitrella patens* (XP_001770447), *Selaginella moellendorfii* (XP_002962016, XP_002960875), *Synechococcus* sp. PCC 7002 (YP_001733693), *Vitis vinifera* (XP_002267374, XP_002266093, XP_002281446).

Interestingly, the occurrence of at least three RIBA homologs is conserved among all angiosperms of which complete genome data are available. The sequence similarity of the different isoforms was investigated by phylogenetic analysis (Figure 6). As indicated, *Selaginella*, an early tracheophyte, represents the first plant harboring more than one *RIBA* gene. Already one of the two isoforms found in the genome of this lycopodiophyte (designated SmRIBA2 in Figure 6) is situated in a RIBA3-specific clade of the phylogenetic tree. A comparison of protein primary sequences reveals an exchange of essential amino acids in all members of this RIBA3 clade and thus implies a loss of DHBPS function in this group early in higher plant evolution (Figure S1). This hints at a specific requirement for an independent GCHII activity that became irreplaceable due to an acquisition of special functions in plant metabolism. Interestingly, evolutionary changes of GCHII activities have been reported recently for three GCHII isogenes in *Streptomyces*[28].

It is noteworthy that *Arabidopsis* possesses an unusual set of RIBA isoforms with AtRIBA2 being monofunctional in riboflavin biosynthesis. All other inspected angiosperm species comprise *RIBA2* genes that encode all necessary amino acids to fulfill DHBPS as well as GCHII function as illustrated by the amino acid alignment depicted in Figure S2. Due to the close relationship of RIBA1 and RIBA2 isoforms they are forming a common clade in the phylogenetic tree. However, the unusual evolutionary situation of AtRIBA2 is reflected by an extended branch length (Figure 6).

At present, we hypothesize that the loss of bifunctionality for two out of three AtRIBA isoforms hints either at novel metabolic functions or at a structural role in the spatial organization of plant riboflavin biosynthesis. To investigate the specific impact of a loss of AtRIBA2 and AtRIBA3 function on plant metabolism is hence a challenging task to be addressed in the future.

## 4. Experimental Section

### 4.1. Generation of Antisense Lines

The AtRIBA1 coding region was amplified using primers 17 and 18 (Table S1). The product was cut using *Sma*I and inserted into binary vector pGL1, which was derived from pGPTV-bar [30] by removing GUS and introducing a 35S CaMV promoter and a multiple cloning site. *A. thaliana* was transformed using standard procedures.

### 4.2. Heterologous Overexpression

Enzymatically essential RIBA regions were identified based on alignments with prokaryotic RibA and RibB ([17], therein Figure S1). *Arabidopsis* sequences were adapted to *E. coli* codon usage and *N*-terminal His-tags integrated (Data S1). Sequences were provided as pUC57 subclones by GenScript (Piscataway, NJ, USA). *Nde*I/*Hin*dIII fragments were cloned into pET22b(+) (Merck, Darmstadt, Germany). Expression in ArcticExpress™ (DE3) RIL Competent Cells (Agilent, Santa Clara, CA, USA) was induced with 1 mM IPTG at 13 °C for 24 h. Soluble recombinant proteins were purified via FPLC using HisTrap HP columns (GE Healthcare, Uppsala, Sweden) and dialyzed with SnakeSkin^TM^ Pleated Dialysis Tubing (10,000 MWCO, Thermo Scientific, Waltham, MA, USA) in 20 mM Tris-HCl, 200 mM NaCl, 5% glycerol, pH 8.4. The concentration of recombinant RIBA protein fractions was determined by comparison to Bovine Serum Albumin standards on Coomassie stained gels.

### 4.3. Assays of GCHII and DHBPS Activity

The enzyme assays were performed according to [31] with minor modifications. For GCHII activity: Assay mixtures (100 μL) containing 100 mM Tris-HCl pH 7.8, 5 mM MgCl_2_, 5 mM DTT, 100 μM GTP and either RibA from *B. subtilis* (0.5 mg/mL) or RIBA1-3 from *A. thaliana* (0.975, 0.15, 0.175 μg/μL) were incubated 1 h at 37 °C. After addition of EDTA (10 mM) and diacetyl (5 mM), the samples were incubated 1 h at 37 °C. Then, 100 μL of TCA (300 mM) were added and samples centrifuged for 2 min at 13,000 rpm and 10 °C. 20 μL from the supernatant were loaded on RP18 column (Lichrospher100 RP18, 5 μL, 250 × 4 mm, flow rate 1 mL min^−1^) and eluted isocratically with methanol/water (*v*/*v* 4:6). Effluent fluorescence of 6,7-dimethylpterin was monitored (λ_ex_ 340 nm; λ_em_ 400 nm). For DHBPS activity: assay mixtures (100 μL) containing 100 mM Tris-HCl pH 7.8, 5 mM MgCl_2_, 5 mM DTT, 100 μM d-ribose 5-phosphate, phosphoriboisomerase (0.5 U/mL), 100 μM, 5-diamino-6-ribitylamino-2,4(1*H*,3*H*) pyrimidinedione, lumazine synthase of *B. subtilis* (0.5 U/mL) and either *B. subtilis* RibA (0.5 mg/mL) or *A. thaliana* RIBA1-3 (0.975, 0.15, 0.175 μg/μL) were incubated 2 h at 37 °C. 20 μL from the supernatant were loaded on RP18 column and eluted isocratically with methanol/water/formic acid (26:234:1). Effluent fluorescence of 6,7-dimethyl-8- ribityllumazine was monitored (λ_ex_ 408 nm; λ_em_ 490 nm). Synthetic dimethylpterin and 6,7-dimethyl- 8-ribityllumazine have been used as calibration standards.

### 4.4. Complementation Assays

*RIBA1* and *RIBA3* sequences were cut from pUC57 (see above) with *Ecl*136II/*Hinc*II (*RIBA1*) or *Ecl*136II/*Sma*I (*RIBA3*). For *RIBA2*, primers 1 and 2 (Table S1) were applied using *RIBA2* in pUC57 as template; the resulting product was cut with *Psi*I. All *RIBA* fragments were inserted into the *Eco*RV site of pACYC184 (NEB, Ipswich, MA, USA). Constructs were transformed into *E. coli* knock-out strains BSV18 (*ribA18::Tn5*; CGSC# 6992) and BSV11 (*ribB11::Tn5*; CGSC# 6991) defective in RibA (GCHII) and RibB (DHBPS), respectively, [32] obtained from the *E. coli* Genetic Stock Center (http://cgsc.biology.yale.edu/).

For growth assays, LB cultures with 0.4 g/L riboflavin were inoculated with an overnight culture and grown at 37 °C, 250 rpm to an OD_600_ of 0.6. The mutants require high concentrations of externally added flavins due to the lack of specific riboflavin import mechanisms in *E. coli*. Cells were pelleted and washed three times in M9 minimal medium containing 20% glucose [33]. M9 cultures containing antibiotics were inoculated with washed cells to give an initial OD_600_ of 0.1.

### 4.5. RNA Isolation and Quantification

Total RNA was isolated using innuPREP Plant RNA Kit (Analytic Jena, Jena, Germany). 0.4 μg of DNAseI-pretreated total RNA was reverse transcribed with oligo dT18 using RevertAid RT (Thermo Scientific, Waltham, MA, USA). cDNA was amplified with SensiMix SYBR No-ROX kit (Bioline, London, UK) on a CFX96 Real-Time System (Bio-Rad, Hercules, CA, USA) using the oligonucleotides listed in Table S1. Expression rates were calculated relative to SAND (At2g28390 [34]) according to the 2^−ΔCT^ method [35,36].

### 4.6. HPLC Analysis

Plant material (0.1 g) harvested from rosette leaves of 8-week-old plants grown under short day conditions (10 h light/14 h dark) at 120 μmole photons m^−2^ s^−1^ was ground in liquid N_2_, resuspended in 0.5 mL of methanol/methylen chloride (9:10), incubated for 2 h under gentle agitation at 4 °C and centrifuged. Samples were analyzed on HPLC system 1100 (Agilent, Santa Clara, CA, USA) using a NovaPak C18 column (150 mm; 3.9 mm diameter; 4 μm particle size) and eluted with a linear gradient of 50 mM ammonium acetate (pH 6) and methanol from 100% to 47% over 20 min at 0.8 mL/min. Riboflavin, FMN and FAD were detected by fluorescence (λ_ex_ 265 nm, λ_em_ 530 nm) and confirmed using authentic standards (Sigma-Aldrich, St. Louis, MO, USA).

### 4.7. Subcellular Localization of RIBA-GFP Fusions

*N*-termini of RIBA proteins were amplified from cDNA using primers 3/4, 5/6 and 7/8 (Table S1). Products were cut by *Sma*I (RIBA1) or *Kpn*I/*Sma*I (RIBA2, RIBA3) restrictions and ligated into modified pCF203. [37] Resulting plasmids were transformed into *A. tumefaciens* pGV2260 and used to infiltrate *Nicotiana benthamiana* leaves [38]. Transient GFP expression was visualized by confocal laser scanning microscopy (CLSM) (λ_ex_ 488 nm, GFP λ_em_ 500–550 nm, chlorophyll λ_em_ 600–700nm).

### 4.8. Protein Analysis

Proteins were separated on 12% polyacrylamide gels as described [39]. Immune-detection of AtRIBA used a polyclonal anti-RIBA1 antiserum (Biogenes, Berlin, Germany) which was affinity-purified on nitrocellulose-bound antigen. Immune-detection used HRP-conjugated anti-rabbit antibody (Sigma-Aldrich, St. Louis, MO, USA), ECL Western Blotting Detection System (GE Healthcare, Uppsala, Sweden) and a Stella 3200 (raytest Isotopenmessgeräte GmbH, Straubenhardt, Germany).

## 5. Conclusions

Converged riboflavin biosynthesis starts with GTP cyclohydrolase II (GCHII) and 3,4-dihydroxy-2-butanone-4-phosphate synthase (DHBPS). Three genes, *AtRIBA*1, *AtRIBA2* and *AtRIBA3* encode the putatively bifunctional RIBA protein with both catalytic properties in *Arabidopsis*. However, two out of three members of the *Arabidopsis* gene family encode the bipartite RIBA protein, but show only one of the two enzymatic functions required for riboflavin biosynthesis. Thus, AtRIBA3 possesses only a GCHII function, while only AtRIBA2 possesses the DHBPS activity. Interestingly, a phenotypical analysis of an Arabidopsis *ribA1* mutant revealed that the two monofunctional RIBA2 and 3 do not compensate for RIBA1 deficiency *in planta*.

## Supplementary Information

Figure S1 Comparison of RIBA3 clade members. Partial alignments of RIBA3 clade members with bifunctional AtRIBA1 (underlined). Enzymatically important amino acid residues for DHBPS (**A**) and GCHII (**B**) function are highlighted in yellow. Substitutions in catalytic or substrate binding domains of the different RIBA3 sequences are marked in red. The loss of essential amino acids is restricted to DHBPS regions. This classifies all members of the RIBA3 clade as monofunctional enzymes possessing GTP cyclohydrolase II activity only. Important residues and domains for both enzymes have been identified previously [40–43]. MULTALIN (http://multalin.toulouse.inra.fr/multalin/) [44] and GeneDoc (http://www.nrbsc.org/gfx/genedoc) [45] were used to generate and edit alignments. Amino acid numbers are indicated at the right margin. At, *Arabidopsis thaliana*; Vv, *Vitis vinifera*; Os, *Oryza sativa*; Sm, *Selaginella moellendorfii*.

Figure S2 Alignment of RIBA1 and RIBA2 isoforms. Sequence comparison of higher plant RIBA1 and RIBA2 (bold) proteins with bifunctional AtRIBA1 (underlined). Enzymatically important amino acid residues for DHBPS (**A**) and GCHII (**B**) function are highlighted in yellow. Deviations in zinc binding residues as well as a *C*-terminal deletion uniquely present in AtRIBA2 are marked in red. The loss of essential amino acids is restricted to the GCHII part of AtRIBA2, qualifying all other RIBA2 isoforms as truly bifunctional proteins.

Data S1 Sequences of synthetic genes AtRIBA1-3. Nucleotide sequences have been modified by avoiding rare codons known to impede expression in *E. coli* and by introduction of unique restriction sites. *N*-terminally a sixfold His motif and an enterokinase cleavage site were added (underlined), restriction sites at the 5′- and 3′-termini are shown in italics. The derived primary protein sequences starting at amino acid 127 (AtRIBA1), 105 (AtRIBA2) and 100 (AtRIBA3), respectively, of the precursor proteins were not altered by the introduced nucleotide changes.

**Table S1:** List of used Primers.

Nr.	Designation	Sequence 5′→3′
1	RibA2_PsiI_fw	AATTATAACAGTCGACGGGCCCG
2	RibA2_PsiI_rev	GCTTATAATACCTCGCGAATGCATCT
3	RibA1_GFP_fw	ACCCGGGACAATGTCTTCCATCAATTTATCC
4	RibA1_GFP_rev	ACCCGGGATCTTCTCTAGAGATCACTGCAG
5	RibA2_GFP_fw	CAGGTACCAAAATGGCGTCGCTTACT
6	RibA2_GFP_rev	ACCCGGGTTCAGGAGAATCCATTGTTG
7	RibA3_GFP_fw	CAGGTACCACGATGATGGATTCTGCTTTA
8	RibA3_GFP_rev	ACCCGGGATCAAACAACGACCCGTC
9	qRT_At5g64300_fw2	TTGTTACTTCTTGTTGTCGGG
10	qRT_At5g64300_rev2	TGATGATCCACATTCCACAC
11	qRT_At2g22450_fw2	GGTTCCACTCATTACTACTCCT
12	qRT_At2g22450_rev2	AAACTAAGTCACTCAAGAAGCC
13	qRT_At5g59750_fw1	AGACTAATGACGAATAACCCTG
14	qRT_At5g59750_rev1	ATATCTTCTGTTCTCCTTGGTG
15	qRT_SAND_fw	AACTCTATGCAGCATTTGATCCACT
16	qRT_SAND_rev	TGATTGCATATCTTTATCGCCATC
17	AtRIBA1fw	ACCCGGGACAATGTCTTCCATCAATTTATCC
18	AtRIBA1rev	ACCCGGGTCAGGACTCAGATTCAGACTCAATC

## Abbreviations

CLSM: confocal laser scanning microscopy
DHBPS: 3,4-dihydroxy-2-butanone-4- phosphate synthase
GCHII: GTP cyclohydrolase II
GFP: green fluorescent protein
