# Supplementary figures and images for: Arabidopsis RIBA Proteins: Two out of Three Isoforms Have Lost Their Bifunctional Activity in Riboflavin Biosynthesis
Source: Int J Mol Sci. 2012 Oct 31;13(11):14086–105. doi: 10.3390/ijms131114086 (PMC3509567; doi:10.3390/ijms131114086)

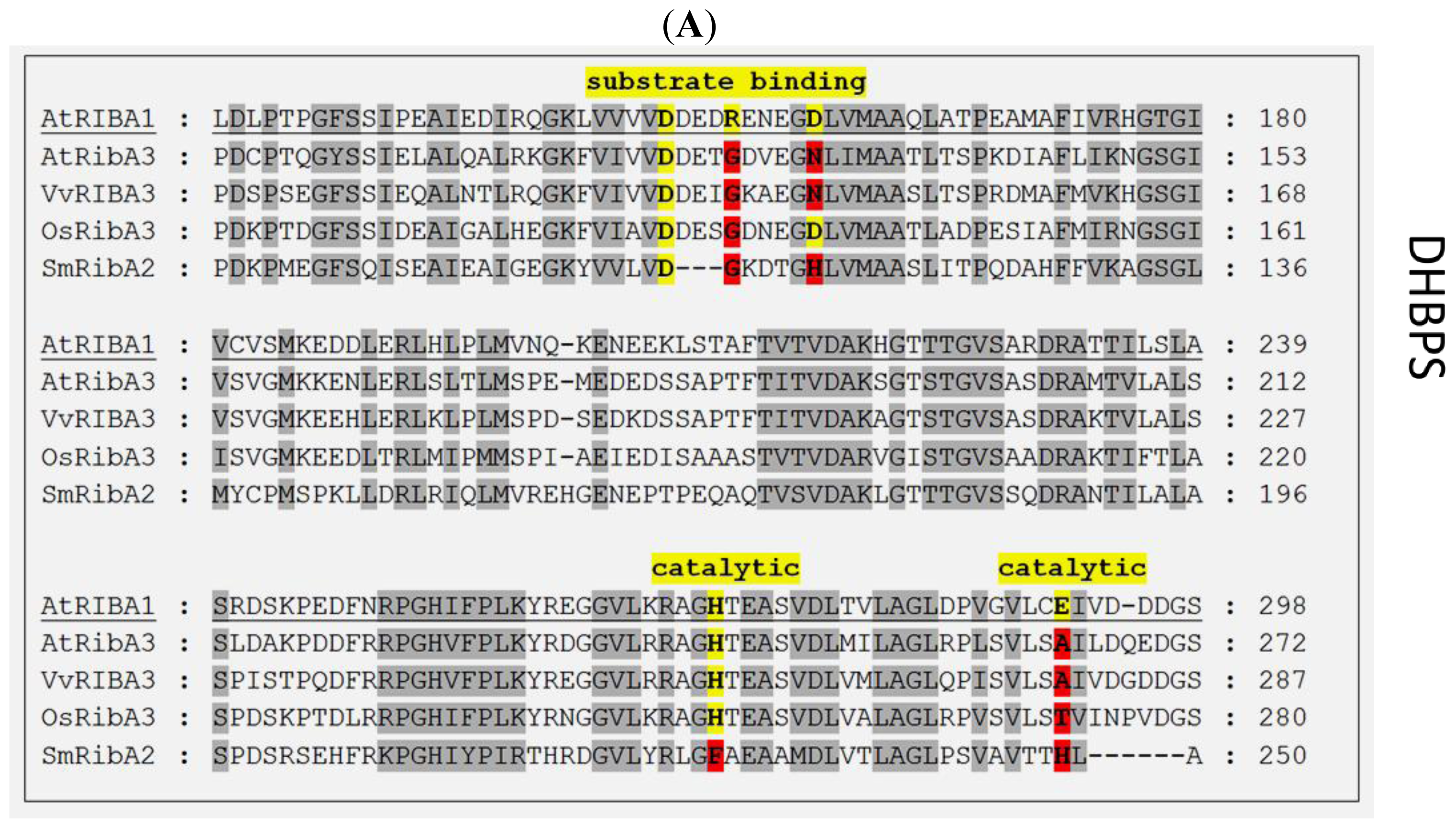

Supplement: Figure S1 — Comparison of RIBA3 clade members. Partial alignments of RIBA3 clade members with bifunctional AtRIBA1 (underlined). Enzymatically important amino acid residues for DHBPS (A) and GCHII (B) function are highlighted in yellow. Substitutions in catalytic or substrate binding domains of the different RIBA3 sequences are marked in red. The loss of essential amino acids is restricted to DHBPS regions. This classifies all members of the RIBA3 clade as monofunctional enzymes possessing GTP cyclohydrolase II activity only. Important residues and domains for both enzymes have been identified previously [40–43]. MULTALIN (http://multalin.toulouse.inra.fr/multalin/) [44] and GeneDoc (http://www.nrbsc.org/gfx/genedoc) [45] were used to generate and edit alignments. Amino acid numbers are indicated at the right margin. At, Arabidopsis thaliana; Vv, Vitis vinifera; Os, Oryza sativa; Sm, Selaginella moellendorfii. [file ijms-13-14086s1a.tif]

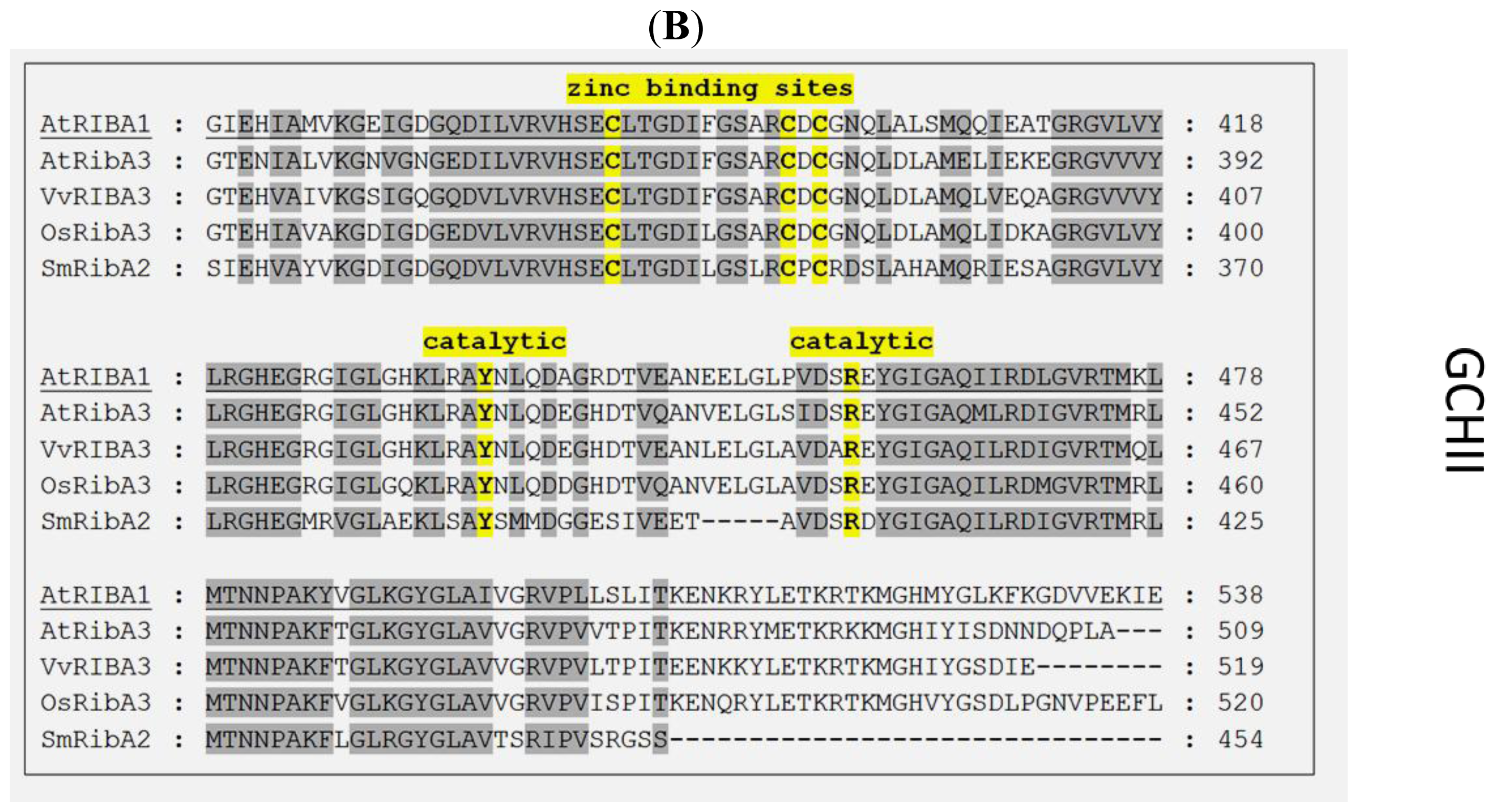

Supplement: Figure S1 — Comparison of RIBA3 clade members. Partial alignments of RIBA3 clade members with bifunctional AtRIBA1 (underlined). Enzymatically important amino acid residues for DHBPS (A) and GCHII (B) function are highlighted in yellow. Substitutions in catalytic or substrate binding domains of the different RIBA3 sequences are marked in red. The loss of essential amino acids is restricted to DHBPS regions. This classifies all members of the RIBA3 clade as monofunctional enzymes possessing GTP cyclohydrolase II activity only. Important residues and domains for both enzymes have been identified previously [40–43]. MULTALIN (http://multalin.toulouse.inra.fr/multalin/) [44] and GeneDoc (http://www.nrbsc.org/gfx/genedoc) [45] were used to generate and edit alignments. Amino acid numbers are indicated at the right margin. At, Arabidopsis thaliana; Vv, Vitis vinifera; Os, Oryza sativa; Sm, Selaginella moellendorfii. [file ijms-13-14086s1b.tif]

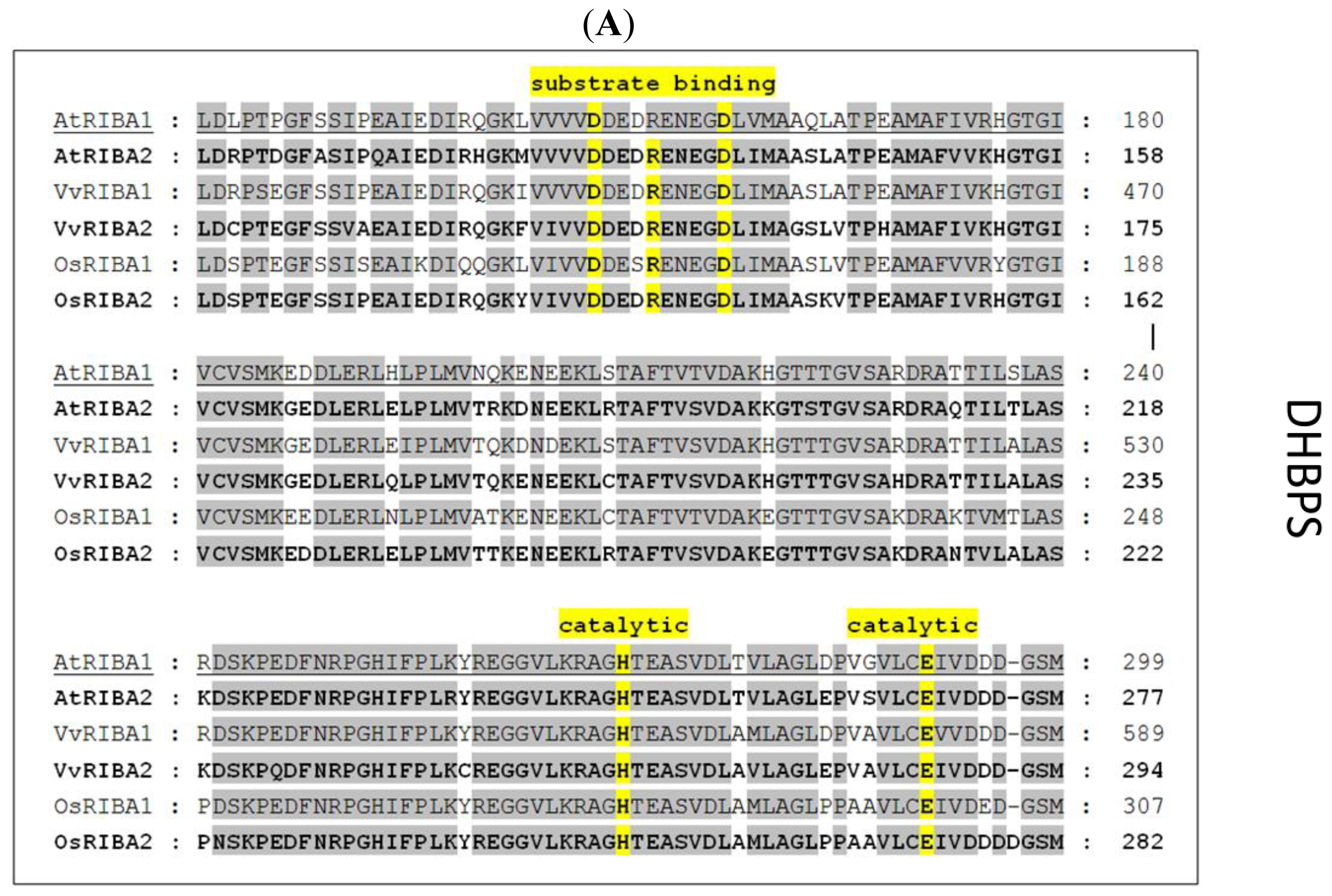

Supplement: Figure S2 — Alignment of RIBA1 and RIBA2 isoforms. Sequence comparison of higher plant RIBA1 and RIBA2 (bold) proteins with bifunctional AtRIBA1 (underlined). Enzymatically important amino acid residues for DHBPS (A) and GCHII (B) function are highlighted in yellow. Deviations in zinc binding residues as well as a C-terminal deletion uniquely present in AtRIBA2 are marked in red. The loss of essential amino acids is restricted to the GCHII part of AtRIBA2, qualifying all other RIBA2 isoforms as truly bifunctional proteins. [file ijms-13-14086s2a.tif]

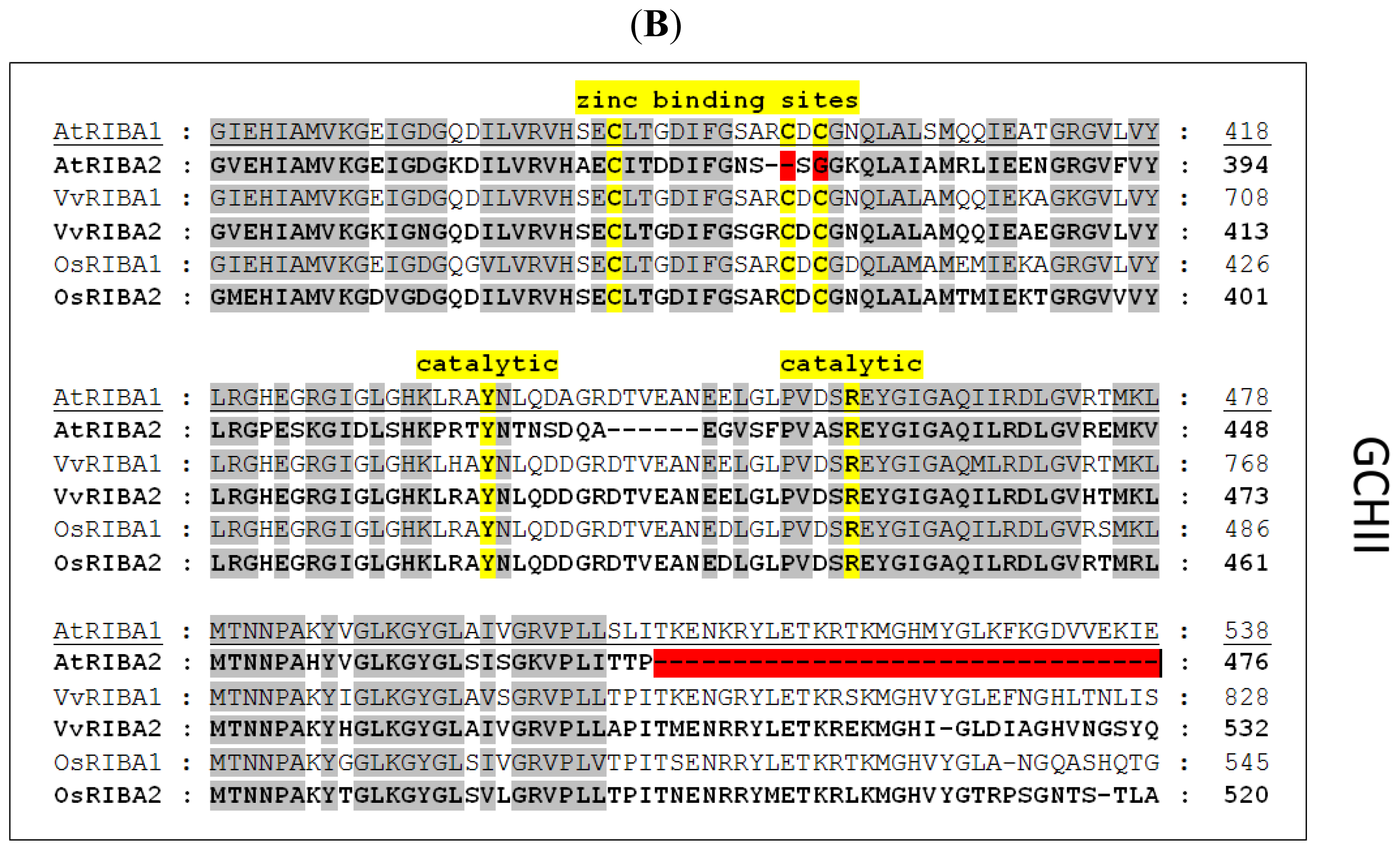

Supplement: Figure S2 — Alignment of RIBA1 and RIBA2 isoforms. Sequence comparison of higher plant RIBA1 and RIBA2 (bold) proteins with bifunctional AtRIBA1 (underlined). Enzymatically important amino acid residues for DHBPS (A) and GCHII (B) function are highlighted in yellow. Deviations in zinc binding residues as well as a C-terminal deletion uniquely present in AtRIBA2 are marked in red. The loss of essential amino acids is restricted to the GCHII part of AtRIBA2, qualifying all other RIBA2 isoforms as truly bifunctional proteins. [file ijms-13-14086s2b.tif]

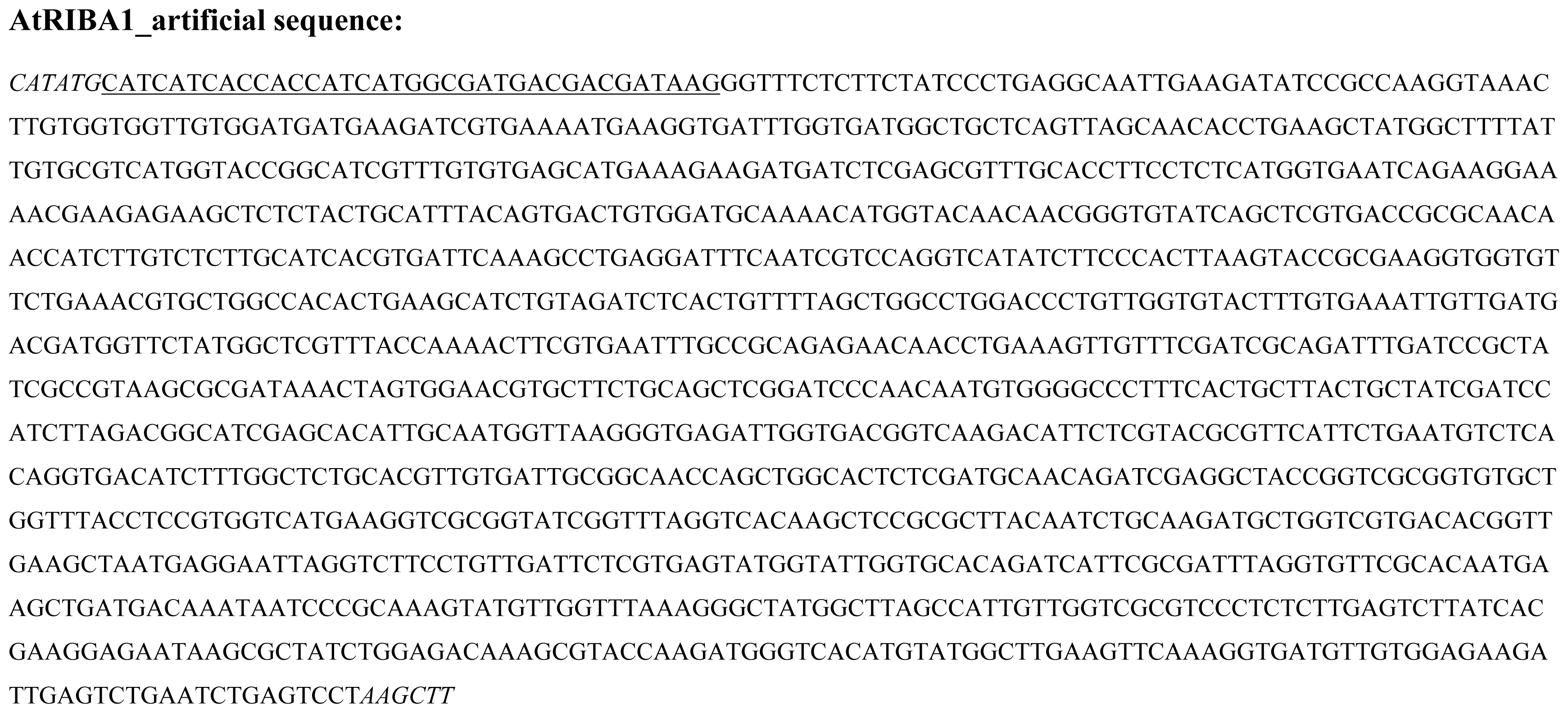

Supplement: Data S1 — Sequences of synthetic genes AtRIBA1-3. Nucleotide sequences have been modified by avoiding rare codons known to impede expression in E. coli and by introduction of unique restriction sites. N-terminally a sixfold His motif and an enterokinase cleavage site were added (underlined), restriction sites at the 5′- and 3′-termini are shown in italics. The derived primary protein sequences starting at amino acid 127 (AtRIBA1), 105 (AtRIBA2) and 100 (AtRIBA3), respectively, of the precursor proteins were not altered by the introduced nucleotide changes. [file ijms-13-14086s3a.tif]

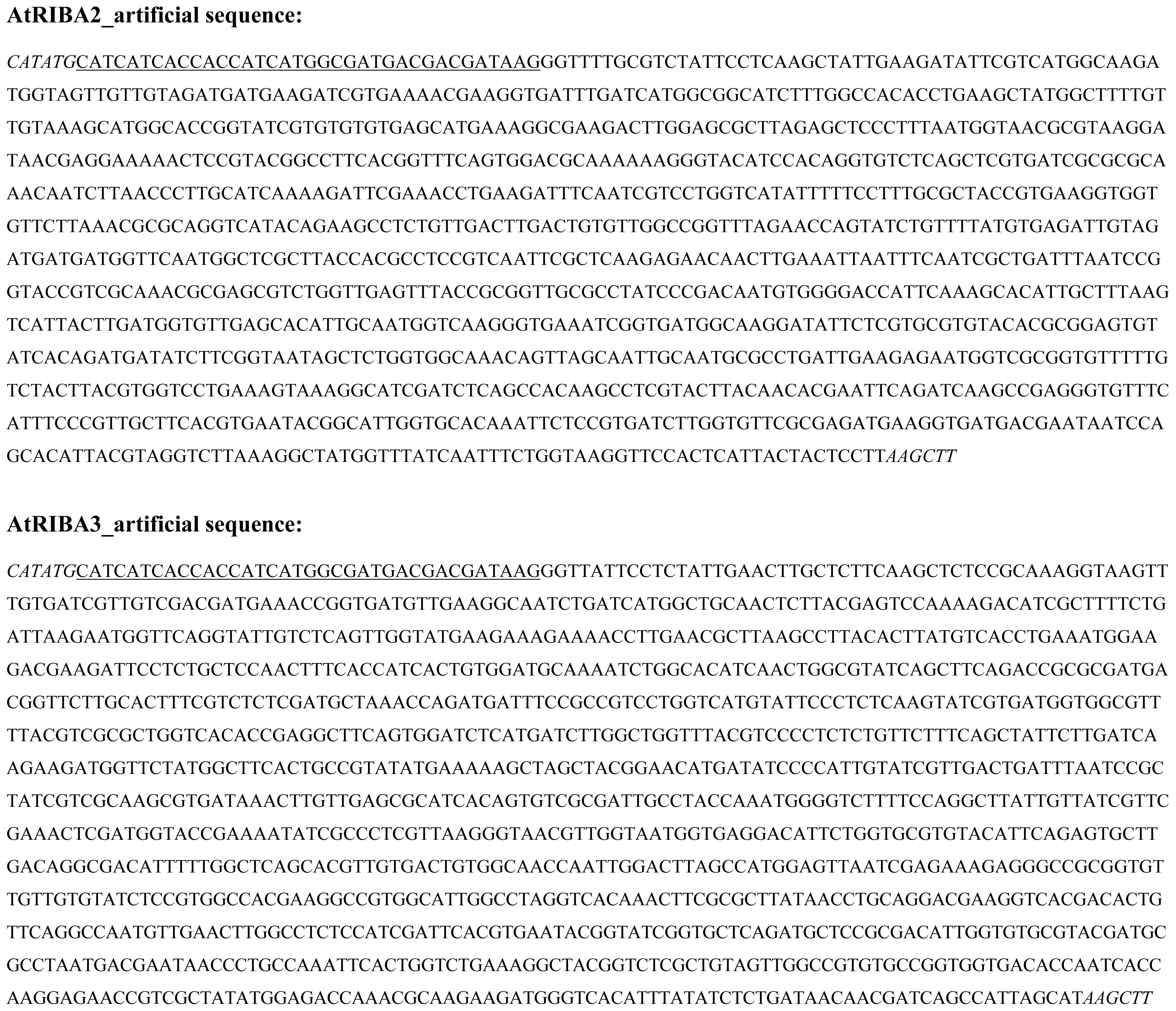

Supplement: Data S1 — Sequences of synthetic genes AtRIBA1-3. Nucleotide sequences have been modified by avoiding rare codons known to impede expression in E. coli and by introduction of unique restriction sites. N-terminally a sixfold His motif and an enterokinase cleavage site were added (underlined), restriction sites at the 5′- and 3′-termini are shown in italics. The derived primary protein sequences starting at amino acid 127 (AtRIBA1), 105 (AtRIBA2) and 100 (AtRIBA3), respectively, of the precursor proteins were not altered by the introduced nucleotide changes. [file ijms-13-14086s3b.tif]
